# The relationship between expectation, experience and perception of labour pain: an observational study

**DOI:** 10.1186/s40064-016-3366-z

**Published:** 2016-10-11

**Authors:** Huseyin Aksoy, Burak Yücel, Ulku Aksoy, Gokhan Acmaz, Turgut Aydin, Mustafa Alparslan Babayigit

**Affiliations:** 1 Department of Obstetrics and Gynecology, Military Hospital, Kayseri, Turkey; 2 Department of Obstetrics and Gynecology, Kanuni Sultan Suleyman Training and Research Hospital, Istanbul, Turkey; 3 Department of Obstetrics and Gynecology, Memorial Hospital, Kayseri, Turkey; 4 Department of Obstetrics and Gynecology, Training and Research Hospital, Kayseri, Turkey; 5 Department of Obstetrics and Gynecology, Acibadem Hospital, Kayseri, Turkey; 6 Department of Public Health, Gulhane Military Medical Academy, Ankara, Turkey

**Keywords:** Labour pain, Pain expectation, Pain management, Visual analogue pain scale, Postpartum pain perception

## Abstract

The present study was aimed to assess the relationship between pain expectation before labour, labour pain and pain perception after the labour. Pregnant women were asked to rate their pain level on a standard continuous visual analogue scale at various time points. Pain expectancy (PE), labour pain (LP) and postpartum pain perception (PPP) scores were calculated. The final study group was composed of 230 pregnant women after exclusions. Mean age of pregnant women was 26.2 ± 5.79. The mean PE, LP, and PPP scores were 70.11 ± 18.82, 75.72 ± 19.2 and 65.84 ± 19.56, respectively. The difference among pain scores was statistically significant (*p* < 0.001). There was a positive correlation between PE and LP or PE and PPP scores (*p* = 0.27 and *p* = 0.21). The correlations were statistically significant (*p* = 0.01 or *p* = 0.01). In addition, there was a positive correlation between LP and PPP scores (*p* = 0.87) and the correlation was statistically significant (*p* = 0.01). This study showed that, if pregnant women had lower expectations of pain before the labour, they indeed experienced lower amount of pain during the labour.

## Background

Childbirth, a milestone in a woman’s life, is one of the most painful events that likely to experience. Labour pain is situation specific, of limited duration, and contrary to many other sources of pain, is not indicative of underlying pathology, but part of a normal physiological process (Lally et al. 2014). It has been showed that ten percentages of all pregnant women experience severe fear of childbirth; this may overshadow the entire pregnancy, can complicate labour, and lead to an increased number of caesarean section (Storksen et al. 2013; Waldenstrom 2003).

The experience of sensory perception event, as labour pain, is entirely subjective and can vary substantially from one person to the next (Coghill et al. 2003; Koyama et al. 2005; Levine et al. 1978). The sensory events matched with environmental cues logically provide a learned historical context, which is crucial for the prediction and processing of future sensations (Coghill et al. 2003). The experience and future predictions about a stimulus lead to individual variations and these are used to interpret afferent information (Koyama et al. 2005).

In the literature, it was claimed that both the subjective experience of pain and pain-related brain activation diminish through the expectations of decreased pain profoundly (Porro et al. 1998). These declines are common and comprise a functionally diverse set of brain regions, including the thalamus, somatosensory cortex, secondary somatosensory cortex, insula, anterior cingulate cortex, prefrontal cortex, and cerebellum. Although their diversity, all these brain regions are known to exhibit activation that is significantly related to the subjective experience of pain (Koyama et al. 2005). Both the subjective expectation of pain magnitude and the magnitude of brain activation supporting a mental representation of impending pain are positively related to the modulation of pain-related activation by expectations (Bennett 1985). Conversely, in a systematic review (McCrea and Wright 1999) it was shown that women’s factual experiences do not correspond to their expectations related to self-control during delivery. Without regard to the pain experienced, more realistic expectations and self-control during labour, seem to be directly associated with greater satisfaction (Bennett 1985). Expectations that are more realistic and consequently, more positive experiences of labour are associated with the participation in antenatal education activities (Levine et al. 1978; Smith et al. 2012).

Labour presents a physical and psychological challenge. The third trimester of pregnancy can be a difficult time emotionally. Fear and apprehension are experienced alongside excitement or their perceptions after childbirth. There emotions both positive and negative will affect the woman’s childbirth experience (Ebirim et al. 2012). Labour although varies with the individual may be the most painful experience, any women may ever encounter (Iliadou 2009). Labour pain determines the experience she has before and this evaluative process is influenced by prior experiences of previous deliveries and current expectations in multiparous. In this study, we aimed to evaluate the relationship between pain expectation (PE) and experience of labour pain (LP) or postpartum pain perception (PPP) after the labour.

## Methods

### Ethical considerations

The study protocol was approved by institutional review board of Erciyes University (2013/483). All pregnant women signed an informed consent form.

### Setting

This prospective cohort study took place at Obstetrics and Gyneacology Department of Kayseri Research and Training Hospital that is one of the biggest tertiary referral hospital with around six thousand deliveries each year in the middle region of Turkey.

### Patient selection

One thousand three hundred and twenty-two pregnant women were admitted to emergency unit between January 2014 and June 2014 for labour. The inclusion criteria were as follows:

1. Pregnant women who were multiparous and older than 18 years old.
2. Pregnant women who had no active labour pain (Such as pregnant who are hospitalized for induction of labour or ruptured membranes without onset of contractions) at admission to the hospital.

All pregnant women who fulfilled the inclusion criteria were invited to participate in the study. Three hundred and thirty-two pregnant women accepted to participate. The study was performed with 230 pregnant women after exclusions.

The exclusion criteria were as follows:

1. Pregnant women who rated their pain level on a continuous 100-mm VAS (Visual analogue scale) different from 0 just before the study were not included in the study.
2. Pregnant women who delivered by instrumental vaginal delivery (e.g. vacuum or forceps) or caesarean section after pain scoring was performed.
3. Pregnant women who received any pharmacological (e.g., opioids, inhaled analgesia) or non-pharmacological (e.g., hypnosis, acupuncture) or invasive methods (e.g. epidural anesthesia) of pain management during labour.

### Pain scores

Pregnant women were asked to rate their pain level on a standard continuous 100-mm visual analogue scale (VAS) to quantify the pain: 0-mm end indicated “no pain” and 100-mm end indicated “the worst pain ever.”

Pain scoring was performed at various time points:

1. *PE score* Pregnant women were asked to rate their labour pain expectations at the time of admission to the hospital.
2. *LP score* Following the third stage of labour; pregnant women were asked to score their pain level experienced during labour.
3. *PPP score* At 12 h after the labour, pregnant women were asked retrospectively to rate their pain level during the labour.

All procedures were performed by the same team to avoid possible operator-dependent factors (counselling, patient preparation, attitude and operative steps during operation, moral and psychological support).

We documented the patients’ demographics: age, parity, body mass index (BMI), number of previous vaginal delivery, time interval from admission to delivery, delivery week and birth weight.

### Statistical analysis

Statistical analyses were performed using the SPSS (Statistical Package for Social Sciences) software version 22.0. Wilcoxon test was used for repeated measures of visual pain scoring of labour. The correlation coefficient and their significance were calculated using the Spearman test. Two-tailed *p* values of <0.05 were considered statistically significant.

## Results

Mean age of pregnant women was 26.20 ± 5.79. Mean body mass index value of pregnant women was 28.39 ± 4.87. Mean number of parity was 3.34 ± 2.34. Baseline demographic characteristics of pregnant women are demonstrated in Table 1.

**Table 1:** Demographic characteristics of participants (n = 230)

Age (years)	26.20 ± 5.79
Body mass index (kg/m^2^)	28.39 ± 4.87
Number of parity	3.34 ± 2.34
Number of previous vaginal delivery	3.31 ± 2.33
Time interval from admission to delivery (h)	8.76 ± 4.76
Delivery week (weeks)	38.54 ± 2.41
Birth weight (g)	3189.57 ± 440.6

Values are expressed as mean ± standard deviation (SD)

The mean PE, LP, and PPP scores were 70.11 ± 18.82, 75.72 ± 19.2 and 65.84 ± 19.56, respectively. The difference among pain scores was statistically significant (*p* < 0.001) (Table 2).

**Table 2:** Pain scores (VAS) at various time intervals of labour (n = 230)

	Pain expectancy (PE) scores	Labour pain (LP) scores	Postpartum pain perception (PPP) scores	*p*
Pain scores (VAS) (mm)	70.11 ± 18.82	75.72 ± 19.2	65.84 ± 19.56	<0.001

*VAS* visual analog scale, values are expressed as mean ± standard deviation (SD)

There was a positive correlation between PE and LP or PPP scores (0.27 or 0.21) and the correlations were statistically significant (*p* = 0.01). PPP scores decreased with the decrement of LP scores. There was a positive correlation between LP and PPP scores (0.87). The correlation was statistically significant (*p* = 0.01). Correlation coefficients for variables and significant correlations are demonstrated in Table 3.

**Table 3:** Correlation coefficients for variables

	Pain expectancy (PE) scores	Labour pain (LP) scores	Postpartum pain perception (PPP) scores
Pain expectancy (PE) Score	1	0.27 (**)	0.21 (**)
Labour pain (LP) score	0.27 (**)	1	0.87 (**)
Postpartum pain perception (PPP) score	0.21 (**)	0.87 (**)	1

(**) Correlation is significant at *p* = 0.01 level (2-tailed)

## Discussion

Pain is highly modifiable by psychological factors, including expectations (Atlas and Wager 2012). The present study was aimed to assess the relationship between PE, LP and PPP. We observed a positive correlation between PE and LP or PE and PPP scores. This study showed that, if pregnant women had lower expectations of pain before the labour, they indeed experienced lower amount of pain during the labour.

Previous research on the relationship between expectations and pain experience shows that expectations about treatments and about painful stimuli can profoundly influence brain and behavioral markers of pain perception (Atlas and Wager 2012). In the literature, it is clear that there are conflicting results about pain expectation and perception and its subsequent effect on labour pain among authors (Lally et al. 2008). Therefore, this study was investigated pain expectations among pregnant and the impact of these expectations on subsequent labour pain. Additionally, we administered the VAS scoring about the perception of labour pain at 12th hour, since the memory of pain may be affected by the time elapsed from the delivery. Expectations of pain during labour in primiparous pregnant women has been reported to be less or more than actual labour pain (Capogna et al. 1996). In the literature, it has been shown that expectations are shaped from prior experience (Linton and Shaw 2011). In this study, we have only considered women with at least one prior experience of vaginal birth. This is to ensure that the participants of the study had previously experienced the pain of vaginal delivery.

In this study, we asked pregnant women to score their pain expectation and experience levels by VAS scoring system in three different time points. The VAS does not assess culture, communication, mood states of pregnant women or other factors that may influence either the perception or the reporting of pain (McCool et al. 2004). Nonetheless, the VAS is widely implemented because it is easy to use, quick to score, and avoids imprecise terminology (Breivik et al. 2000). In the literature, the VAS is sensitive to acute pain at mild, moderate, and severe levels of intensity and has acceptable reliability in a variety of patients with acute pain such as labour pain (Winkelman et al. 2008).

Self-control during labour that is maintained by women’s ability has been seen as essential for a good birthing experience (Niven and Murphy-Black 2000; Shaban et al. 2014). We found that there was a positive correlation between pre-labour pain expectation and pain during labour. Additionally, there was strong and positive correlation between pain during and after labour. However, pregnant women were reported significantly lower pain scores 12 h after labour than during labour. One way to interpret the present findings is to conclude that all phases of labour are related to each other. Additionally, we are of the opinion that labour pain may be easily forgettable, and this situation begins just after labour.

Women’s ability and accuracy in recalling labour pain are widely debated. Various factors might affect the quality of women’s pain memories. The first and most important of these factors is the presence of a “halo effect” immediately after childbirth. This is where the happiness and reward of holding a healthy baby color the memory of the preceding pain and pushes all the previous pain to the back of our minds as if forgotten (Bennett 1985).

Pain is only one element of the overall birth experience. Memory for pain is influenced by all the other parts of the birth experience. These other factors that contribute to how birth is remembered include satisfaction with health care providers, level of medical intervention, choice and use of pain relief options, the health of the new-born, whether there were any complications, and all sorts of personal factors. All these factors together can play a significant role in determining how pain is remembered. When all of these elements added up to a positive overall birth experience, women reported less pain at the time and were more likely to lower their rating of the pain over time. When these aspects combined with a negative experience, however, women reported more pain in childbirth and did not forget the intensity of labour pain up to 5 years later (Niven and Murphy-Black 2000; Shaban et al. 2014; Smith et al. 2012).

Studies related to women’s memory of labour and delivery have generally concluded that there is significant individual variation in women’s recollection of labour pain. Studies related to women’s memory of labour and birth have generally concluded that women’s recall of labour pain significantly depends on individual variations. In one of these studies, Waldenström et al. (2003) reported that over time, many women remember labour and birth pain as being less severe than they originally recalled. Another study conducted in 1383 women who provided complete data up to 5 years after the birth showed that memory of labour pain declines during the observation period but not in women with a negative overall experience of childbirth (Waldenstrom and Schytt 2009). Our results are similar to the ones in the literature. Women reported significantly lower pain score in the postpartum period (12 h after the birth) according to labour pain in our study. These findings suggest that women tend to reduce their rating of the pain over time. Therefore, preparing women psychologically and socially for childbirth could help most of them how their memory of labour and birth are shaped.

We recognize several limitations in our study. Pain is a difficult outcome to measure due to its subjective and multifaceted nature. VAS pain scoring system could be insufficient to determine these differences. Other limitations of the present study reside in its single-center design as well as small sample size and limited demographic data. The study is strengthened by its prospective nature and being a study that observed the relationship between all three components of labour pain.

## Conclusions

In this study, we demonstrated that if women had lower expectations of pain before the labour, they indeed experienced lower amount of pain during the labour. Thus, a rationalist approach to reducing the pain experience during labour should aim to decrease pain expectations of pregnant before the new experience. There is also a need for further, larger scale studies including relationship between expectations and experiences during labour interacting with environmental factors and development of pain.

## Abbreviations

PE: Pain expectancy
LP: Labour pain
PPP: Postpartum pain perception
VAS: Visual analogue scale

## References

[CR1] Atlas LY, Wager TD (2012). How expectations shape pain. Neurosci Lett.

[CR2] Bennett A (1985). The birth of a first child: do women’s reports change over time?. Birth.

[CR3] Breivik EK, Bjornsson GA, Skovlund E (2000). A comparison of pain rating scales by sampling from clinical trial data. Clin J Pain.

[CR4] Capogna G, Alahuhtat S, Celleno D, De Vlieger H, Moreira J, Morgan B, Moore C, Pasqualetti P, Soetens M, Van Zundertl A, Vertommen JD (1996). Maternal expectations and experiences of labour pain and analgesia: a multicentre study of nulliparous women. Int J Obstet Anesth.

[CR5] Coghill RC, McHaffie JG, Yen YF (2003). Neural correlates of interindividual differences in the subjective experience of pain. Proc Natl Acad Sci USA.

[CR6] Ebirim LN, Buowari OY, Ghosh S (2012) Physical and psychological aspects of pain in obstetrics. In: Ghosh S (ed) Pain in perspective. InTech

[CR7] Iliadou M (2009). Labour pain and pharmacological pain relief practice points. Health Sci J.

[CR8] Koyama T, McHaffie JG, Laurienti PJ, Coghill RC (2005). The subjective experience of pain: where expectations become reality. Proc Natl Acad Sci USA.

[CR9] Lally JE, Murtagh MJ, Macphail S, Thomson R (2008). More in hope than expectation: a systematic review of women’s expectations and experience of pain relief in labour. BMC Med.

[CR10] Lally JE, Thomson RG, MacPhail S, Exley C (2014). Pain relief in labour: a qualitative study to determine how to support women to make decisions about pain relief in labour. BMC Pregnancy Childbirth.

[CR11] Levine JD, Gordon NC, Fields HL (1978). The mechanism of placebo analgesia. Lancet.

[CR12] Linton SJ, Shaw WS (2011). Impact of psychological factors in the experience of pain. Phys Ther.

[CR13] McCool WF, Smith T, Aberg C (2004). Pain in women’s health: a multi-faceted approach toward understanding. J Midwifery Womens Health.

[CR14] McCrea BH, Wright ME (1999). Satisfaction in childbirth and perceptions of personal control in pain relief during labour. J Adv Nurs.

[CR15] Niven CA, Murphy-Black T (2000). Memory for labor pain: a review of the literature. Birth.

[CR16] Porro CA, Cettolo V, Francescato MP, Baraldi P (1998). Temporal and intensity coding of pain in human cortex. J Neurophysiol.

[CR17] Shaban I, Mohammad K, Homer C (2014). Development and validation of women’s satisfaction with hospital-based intrapartum care scale in Jordan. J Transcult Nurs.

[CR18] Smith CA, Levett KM, Collins CT, Jones L (2012). Massage, reflexology and other manual methods for pain management in labour. Cochrane Database Syst Rev.

[CR19] Storksen HT, Garthus-Niegel S, Vangen S, Eberhard-Gran M (2013). The impact of previous birth experiences on maternal fear of childbirth. Acta Obstet Gynecol Scand.

[CR20] Waldenstrom U (2003). Women’s memory of childbirth at two months and one year after the birth. Birth.

[CR21] Waldenstrom U, Schytt E (2009). A longitudinal study of women’s memory of labour pain–from 2 months to 5 years after the birth. BJOG Int J Obstet Gynaecol.

[CR22] Winkelman C, Norman D, Maloni JA, Kless JR (2008). Pain measurement during labor: comparing the visual analog scale with dermatome assessment. Appl Nurs Res.

